# Fluorescent in-situ hybridization (FISH) for BCR/ABL in chronic myeloid leukemia after bone marrow transplantation

**DOI:** 10.1590/S1516-31802001000100005

**Published:** 2001-01-02

**Authors:** Maria de Lourdes Lopes Ferrari Chauffaille, José Salvador Rodrigues Oliveira, Maura Romeo, José Kerbauy

**Keywords:** BCR/ABL Genes, Chronic myeloid leukemia, Fluorescent in-situ hybridization, Genes BCR/ABL, Leucemia mielóde crônica, Hibridação *in situ* por fluorescência, Leucemia mielóide crônica

## Abstract

**CONTEXT:**

Identification of Philadelphia chromosome or BCR/ABL gene rearrangement in chronic myeloid leukemia is important at diagnosis as well as after treatment.

**OBJECTIVE:**

To compare the results of karyotyping using fluorescent in-situ hybridization (FISH) upon diagnosis and 1 year after bone marrow transplantation in 12 patients.

**TYPE OF STUDY:**

Diagnostic test and residual disease detection.

**SETTING:**

Hematology and Hemotherapy Department, Federal University of São Paulo/Escola Paulista de Medicina, São Paulo, Brazil.

**SAMPLE:**

12 patients with chronic myeloid leukemia at diagnosis and 1 year after bone marrow transplantation.

**DIAGNOSTIC TEST:**

Karyotyping was done in the usual way and the BCR/ABL gene-specific probe was used for FISH.

**MAIN MEASUREMENTS:**

Disease at diagnosis and residual.

**RESULTS:**

At diagnosis, 10 patients presented t(9;22)(q34.1;q11) as well as positive FISH. Two cases did not have metaphases but FISH was positive. After bone marrow transplantation, 8 patients presented normal karyotype, 1 had persistence of identifiable Philadelphia chromosome and 3 had no metaphases. Two cases showed complete chimera and 2 had donor and host cells simultaneously. FISH was possible in all cases after bone marrow transplantation and confirmed the persistence of identifiable Philadelphia chromosome clone in one patient, and identified another that did not present metaphases for analysis. Cases that showed mixed chimera in karyotype were negative for BCR/ABL by FISH.

**CONCLUSION:**

The applicability of FISH is clear, particularly for residual disease detection. Classical and molecular cytogenetics are complementary methods.

## RESUMO

**CONTEXTO:**

Na leucemia mielóide crônica, a detecção do cromossomo Philadelphia ou o rearranjo gênico BCR/ABL é importante tanto ao diagnóstico como após o tratamento.

**OBJETIVO:**

Comparar os resultados do cariótipo com a hibridação *in situ* por fluorescência ao diagnóstico e, após um ano de transplante de medula óssea, em 12 pacientes com leucemia mielóide crônica.

**TIPO DE ESTUDO:**

Teste diagnóstico e detecção de doença residual.

**LOCAL:**

Disciplina de Hematologia e Hemoterapia da Universidade de São Paulo/Escola Paulista de Medicina.

**AMOSTRA:**

12 pacientes com leucemia mielóide crônica ao diagnóstico e um ano após transplante de medula óssea.

**TESTE DIAGNÓSTICO:**

A análise do cariótipo (forma clássica) e a hibridação *in situ* por fluorescência com a sonda específica para os genes BCR/ABL.

**VARIÁVEIS ESTUDADAS:**

Doença ao diagnóstico e residual.

**RESULTADOS:**

Ao diagnóstico, 10 pacientes apresentaram t(9;22)(q34.1;q11) no cariótipo assim como pela hibridação *in situ* por fluorescência. Dois casos em que na citogenética não havia mitoses, apresentaram rearranjo pela hibridação *in situ* por fluorescência. Após o transplante de medula óssea, o cariótipo mostrou ausência do cromossomo Philadelphia em 8 casos, persistência em um e ausência de metáfases em três. Em dois casos havia quimera completa e em outros dois havia concomitância de células do doador e do receptor. A hibridação *in situ* por fluorescência foi possível em todos os casos após o transplante de medula óssea, confirmando a persistência do clone cromossomo Philadelphia num paciente em que o cariótipo também havia mostrado e identificando noutro em que não havia metáfases. Os casos de quimera mista foram negativos para BCR/ABL pela hibridação in situ por fluorescência.

**CONCLUSÃO:**

Ficou evidente a aplicabilidade da hibridação *in situ* por fluorescência, particularmente na detecção de doença residual. A citogenética clássica e a molecular são métodos complementares.

## INTRODUCTION

Chronic myeloid leukemia (CML) is a clonal stem cell disorder characterized by myeloproliferation and by the presence of Philadelphia (Ph) chromosome.

Ph chromosome was the first cytogenetic abnormality described in cancer.^1^ Later it was shown that Ph was a balanced translocation between chromosomes 9 and 22. At a molecular level, there is rearrangement of the BCR and ABL genes with the function of codifying a fusion protein with increased and deregulated tyrosine kinase activity.^2-3^

With the diverse methods available today, Ph-negative patients seem to have poorer survival and evolve more rapidly to blast crisis. However, some authors consider that these patients could have a different disease.^4^

The usual method for detection of Ph is bone marrow G-banding karyotyping. Around 90% of the patients have the translocation detected by this method. Half of the remaining patients may present the rearrangement by fluorescent in situ hybridization (FISH) or reverse transcriptase polymerase chain reaction (PCR).^4^

Nowadays, CML treatment is done using myelosuppressive drugs, but the only way to achieve a cure is bone marrow transplantation (BMT). However, this is possible only for those that have an HLA-compatible donor and are younger than the limiting age for the procedure. Around 50 to 60% of the transplanted patients are cured while the remaining ones relapse or die due to complications.^5^

In the post-BMT period it is necessary to check on the disappearance of the malignant clone using sensitive methods, since the detection of possible residual disease may indicate the need for additional therapy.

The present work aimed to compare the results of karyotyping and FISH at diagnosis and after BMT for detection of residual disease.

## METHODS

Twelve patients with CML were studied at diagnosis and one year after BMT. The aspirated bone marrow was divided and placed into two culture flasks with RPMI 1640 medium, pH 7.2, 20% fetal calf serum, 1% antibiotics and Lglutamine (100mL). Colcemid was added during the last 60 minutes followed by hypotonic treatment with KCl (0.075 mol/L) for 20 min and fixation with Carnoy's fixative (methanol and acetic acid, 3:1) repeated 4 times. The slides were banded for trypsin G-banding (GTG). Ten metaphases from each culture were analyzed and chromosomal abnormalities described in accordance with ISCN 1995.^6^

A portion of the cell pellets stored in Carnoy's fixative was separated for FISH. This material was centrifuged again and resuspended; the slides were made using cytospin (Incibrás). FISH was made following the probe supplier's instructions (BCR/ABL- Oncor). The slides were analyzed through a fluorescent microscope (Olympus BMX60) with DAPI, rhodamine, FITC and triple bandpass filters (Chroma Technology). The best images were captured via a CCD camera mounted on the microscope and linked to a computer with karyotyping and FISH software (CytoVision - Applied Image). Slides showing more than 50% cells with fluorescent dots were selected for analysis. From each slide, at least 200 cells were counted, all of them isolated to avoid overlapping signals. Red dots (rhodamine) corresponded to the ABL (9q34.1) gene and green dots (fluorescein) to the BCR (22q11) gene, so when one cell with two isolated red and green dots was seen, it was counted as normal, without rearrangement. When a cell with one isolated red dot, one isolated green dot and one fused red and green signal was seen, this was considered as presenting rearrangement (Figure). All slides were analyzed by at least two observers. As the difference between the observers presented small variation the results were summed and averaged. Five samples of normal marrow donors served as a control group. The controls provided an estimation of overall hybridization quality of the test material. Using the method of “rate obtained in the control samples plus two standard deviations”, the cutoff level for rearrangement was established as 10%.

**Figure:**
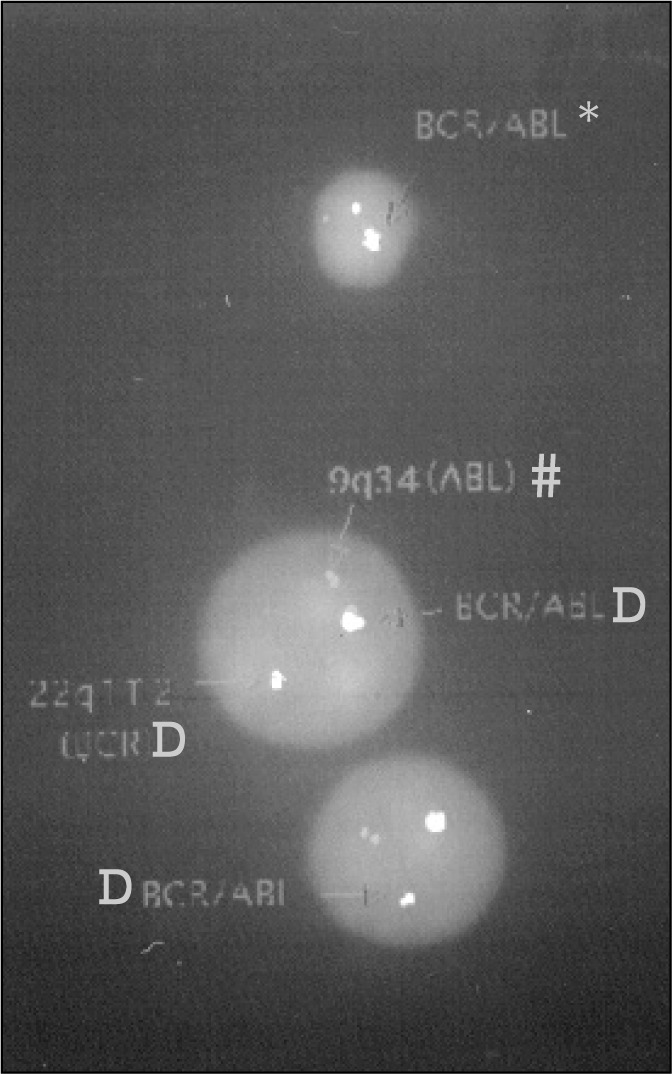
Image of interphase FISH with BCR/ABL probe.

D - *ABL gene*

* - BCR

*# - fusion of* D *and* * *signals or BCR/ABL rearrangement.*

## RESULTS

Table shows patients’ sex, age, date of diagnosis, karyotype at diagnosis, FISH at diagnosis, date of BMT, donor's sex, and karyotype and FISH at one year after BMT.

**Table:** Description of sex, age, date of diagnosis, karyotype and FISH at diagnosis, donor's sex, and karyotyping and FISH at one year after BMT

Case	Sex	Age	Diagnosis			Donor sex	1 year after BMT	
			Date	Karyotype	Fish		Karyotype	Fish
1	F	25	7/92	46,XX,t(9;22)(q34.1;q11)[20]	95%	M	46,XX,t(9;22)[6]/46,XY[13]	25%
2	M	29	11/93	46,XY,t(9;22)(q34.1;q11)[20]	62.5%	M	46,XY[17]	3%
3	F	20	6/93	46,XX,t(9;22)(q34.1;q11)[20]	89%	F	Without metaphases	6%
4	M	42	10/94	46,XY,t(9;22)(q34.1;q11)[20]	76%	F	46,XX[15]	7%
5	F	14	4/97	46,XX,t(9;22)(q34.1;q11)[20]	92%	M	46,XY[16]/46,XX[1]	6%
6	M	30	10/94	46,XY,t(9;22)(q34.1;q11)[20]	WM	M	Without metaphases	7%
7	M	26	4/97	46,XY,t(9;22)(q34.1;q11)[20]	66%	M	46,XY[15]	9%
8	M	26	10/96	46,XY,t(9;22)(q34.1;q11)[20]	WM	F	46,XX[19]	10%
9	M	23	10/96	46,XY,t(9;22)(q34.1;q11)[20]	95%	M	Without metaphases	94%
10	M	42	8/96	46,XY,t(9;22)(q34.1;q11)[20]	98%	M	46,XY[20]	5%
11	F	33	1/96	Without metaphases	71%	F	46,XX[15]	6%
12	F	21	9/96	Without metaphases	68%	M	46,XY[19]/46,XX[1]	8.5%

*M= male, F= female. WM = without material.*

It was possible to compare the results of karyotyping and FISH at diagnosis and one year after transplantation. At diagnosis, 10 patients presented Ph chromosome by cytogenetics and two (11 and 12) did not show metaphases that could be studied. FISH was positive in all except 2 (6 and 8), whose samples did not have enough cells for analysis. Cases that did not present cytogenetic results (11 and 12) presented positive BCR/ABL rearrangement by FISH.

The percentage of cells with BCR/ABL rearrangement by FISH at diagnosis varied from 66 to 98%, with a mean of 81.25%.

One year after transplantation, karyotyping was not possible in 3 cases (3, 6 and 9) due to lack of metaphases. In eight cases (2, 4, 5, 7, 8, 10, 11 and 12), the karyotype was normal while one patient (1) had persistence of the Ph chromosome. Two cases (4 and 8) showed complete chimera, as the karyotype changed to the donor's pattern (different sexes). In the remaining cases in which donor and receptor had the same sex (2, 5, 7, 10 and 11), Ph was not observed. Two cases (5 and 12) presented two cell lines, i.e. donor and host residual cells.

FISH at one year after transplantation was possible in all cases, even in those that did not present metaphases for karyotyping. Case 1 had around 30% of Ph-positive cells, in karyotype as well as FISH. Case 9 had clinical and hematological relapse, a karyotype without metaphases and FISH with 94% of cells with BCR/ABL rearrangement, confirming disease relapse in the chronic phase.

In cases whose karyotype showed persistence of host residual cells even at low percentages, FISH showed absence of rearrangement.

## DISCUSSION

Determination of the presence of the Ph chromosome by karyotyping at diagnosis is necessary as part of differential diagnosis and as prognosis. This is an entirely manual test with a turnaround time of 7 to 15 days. Cheaper and faster methods would be ideal. FISH is a feasible technique, faster (usually 48 hrs) but still expensive due to the cost of probes.

Would there be advantages in replacing karyotype detection of Ph by FISH at diagnosis? The initial answer is no, since karyotyping and FISH were equal, as shown in the present work, except for the fact that FISH was faster. In cases that did not present metaphases by karyotyping, FISH was extremely useful in providing a result. In daily practice, FISH can be very helpful since it may be done on the same sample used for karyotyping without the need for new marrow aspiration. Another reason for continuing with classical cytogenetics at diagnosis is the possibility of detecting additional karyotype abnormalities or variant Ph. This was not seen in the present sample but has been in others.^7^

After treatment, methods that promptly, sensitively and reliably detect residual disease are chosen. Karyotyping has been the classical ideal, but more sensitive techniques may tend to replace it. This in part due to the low sensitivity index of karyotyping, since usually 20 to 25 metaphases are studied and in the post BMT period, when the patient is receiving myelotoxic drugs, there may not be enough metaphases for analysis. The FISH technique is especially valuable in these situations, as it is more sensitive even though it is faster. RT-PCR is still more sensitive but its clinical meaning has to be clarified.^5^

In the present work the BCR/ABL rearrangement was demonstrated one year after BMT in one patient (case 9) whose karyotyping was unsuccessful. Similarly, another case (case 1) that had persistence of Ph in karyotyping and has already been discussed elsewhere,^8^ presented a more confident count in 200 interphases using FISH. This type of information given by FISH is already well known and appreciated.^9^

Cases in which a double population of cells is observed by karyotyping, i.e. donor and host cells, could indicate that residual cloning was not being seen since karyotype sensibility was low. FISH allowed us to conclude that such cases had mosaic without residual disease, at least at the sensitivity level of FISH, and that these data were compatible with clinical aspects. This situation had been observed before in a patient that had 40% of host cells persisting for longer after BMT but with negative PCR and FISH for BCR/ABL rearrangement.^10^ These data confirm the impression that there may be mosaic without relapse.^11^ One year after BMT, Polka et al.^12^ also detected mixed chimera with host residual cells varying from 0.2 to 90%.

The percentage of cells with rearrangement seen using FISH at diagnosis varied from 66 to 98%. Other authors have described levels from 30 to 98% but usually above 77 to 88%.^9,13^ Some cases presented a low percentage of positive interphases due to a low hybridization index but still within acceptable limits (more than 50% of cells with hybridization).

FISH allows later study using stored samples that is valuable. The fact that fixed cells were being utilized was useful in this work, although there were some limitations (cases 6 and 8). Many samples from other patients that fitted the aims of this work were not included, often because the stored material was not in an adequate condition or there were not enough interphases for analysis.

After treatment, some patients presented levels very close to normality (case 7 and 8). These were reevaluated by PCR and until now have remained negative. Periodic examination will allow detection of residual mass alterations.

## CONCLUSION

It was possible to compare the two methods and it is clear that FISH has a well-defined use. FISH and karyotyping are not mutually exclusive methods but complementary.
